# Incidence and prevalence of multiple sclerosis in Hungary based on record linkage of nationwide multiple healthcare administrative data

**DOI:** 10.1371/journal.pone.0236432

**Published:** 2020-07-27

**Authors:** Anna Iljicsov, Dániel Milanovich, András Ajtay, Ferenc Oberfrank, Mónika Bálint, Balázs Dobi, Dániel Bereczki, Magdolna Simó

**Affiliations:** 1 Department of Neurology, Semmelweis University, Budapest, Hungary; 2 MTA-SE Neuroepidemiological Research Group, Budapest, Hungary; 3 Institute of Experimental Medicine, Budapest, Hungary; 4 Centre for Economic and Regional Studies, Budapest, Hungary; 5 Department of Probability Theory and Statistics, Eötvös Loránd University, Budapest, Hungary; University of Catania, ITALY

## Abstract

**Objectives:**

As there were only regional studies in Hungary about the prevalence of multiple sclerosis (MS), we aimed to estimate its epidemiological features using data of Hungary’s single-payer health insurance system.

**Methods:**

Pseudonymized database of claims reported by hospitals and outpatient services between 2004–2016 was analyzed and linked with an independent database of outpatient pharmacy refills between 2010–2016. We established an administrative case definition of MS and validated it on medical records of 309 consecutive patients. A subject was defined as MS-patient if received MS diagnosis (International Classification of Diseases, 10th edition, code G35) on three or more occasions at least in 2 calendar years and at least once documented by a neurologist. Patients were counted as incident cases in the year of the first submitted claim for MS. We allowed a 6-year-long run-in period, so only data between 2010–2015 are discussed.

**Results:**

Sensitivity of the administrative case definition turned out to be 99%, while specificity was >99%. Crude prevalence of MS has increased from 109.3/100,000 in 2010 to 130.8/100,000 in 2015 (*p*-value = 0.000003). Crude incidence declined from 7.1/100,000 (2010) to 5.4/100,000 (2015) (*p*-value = 0.018). Direct standardization − based on European standard population and results of nationwide Hungarian census of 2011 − revealed that age standardized prevalence was 105.2/100,000 (2010), which has grown to 127.2/100,000 (2015) (*p*-value = 0.000001). Age standardized incidence rate declined from 6.7/100,000 (2010) to 5.1/100,000 (2015) (*p*-value = 0.016). The ratio of MS-patients receiving ≥1 prescription for disease modifying treatment increased from 0.19 (2010) to 0.29 (2015) (*p*-value = 0.0051). The female/male ratio of prevalent cases remained 2.6.

**Discussion:**

The prevalence of MS in Hungary is higher than previously reported, the incidence rate is moderate. The prevalence is rising, the incidence rate shows decline. The proportion of patients receiving disease modifying treatment grows but was still around 30% in 2015.

## Introduction

Multiple sclerosis (MS) is a chronic, inflammatory disease of the central nervous system, affecting mostly young adults and possibly leading to irreversible physical, psychical and cognitive disability with a negative impact on quality of life and productivity of patients. After traumatic injury, it is the second most common cause of permanent disability in young adults [1].

According to worldwide estimates of the Multiple Sclerosis International Federation and the World Health Organisation published in Atlas of MS 2013 [2, 3], it affects 2.3 million individuals worldwide and around 690,000 in Europe. The Atlas also states that substantial inequalities exist among regions and countries regarding the access to neurological care, magnetic resonance imaging and disease-modifying treatment. In MS Barometer 2015 [4], published by European Multiple Sclerosis Platform, these inequalities were confirmed even among European countries and also it was underlined that only between 0–75% of MS-patients work in full-time job and only 0–50% of MS-patient have part-time employment (depending on the country). Continuous treatment and complex management of MS is a burden for the health care system and caregivers as well [5, 6] and in the era of upcoming expensive immunomodulatory drugs, optimal allocation and planning of healthcare resources require accurate data on the number of patients affected by the disease.

Its prevalence seems to increase [3, 5], which can only partly be explained by recent modifications of diagnostic criteria [7, 8], improved availability of diagnostic facilities and longer survival of patients. Other suspected causes include increased occurrence of obesity and cigarette consumption in women [9], and changes in lifestyle with diminished exposure to sunlight and vitamin D deficiency [10].

As of today, only regional studies were conducted in Hungary on the epidemiology of MS [11–16], therefore the aim of our present study was to estimate the prevalence and incidence of patients living with MS in the whole country. Given the lack of a national MS-registry, we have used anonymized administrative data supplied by the National Health Insurance Fund (NHIF). Among the advantages of this method are that it covers practically the total population, is cost-effective and the available data are standardized and easy to process. However, it has its limitations, like the risk of suboptimal data quality and impossibility of determining detailed individual clinical information on patients.

We have established an administrative case definition of MS–see the details below–and validated it on a cohort of patients of the Department of Neurology, Semmelweis University, Budapest. Given the high concordance between administrative and clinical classification of non-MS and MS-patients, we applied this case definition on the nationwide database and analyzed the number of MS-patients yearly, determining prevalence and incidence of MS in Hungary.

## Methods

### Population and healthcare system

Hungary is part of the European Union, located in Central Eastern Europe with almost 10 million residents (5,219,149 females and 4,718,479 males according to the latest nationwide census held in 2011) [17].

The country has a universal, single-payer state health insurance system, which covers the total resident population. Since 1996, each individual is assigned a unique nine-digit personal code number (social security identifier) for lifetime, which is used in all public healthcare services, including hospitalization, outpatient specialist care, general practitioner, prescription and pharmacy dispensation of medicine, laboratory and other diagnostic examinations. Service providers submit monthly reports for reimbursement purposes to the NHIF, which is responsible for archiving and processing of these electronic data.

Though in 2009 eighty-eight outpatient centers and 97 hospitals provided neurological outpatient services in the country [18], the complex management of MS-patients is organized in 32 MS-centers, and prescription of MS-specific disease-modifying drugs (DMD) is only authorized for neurologists affiliated to one of these centers. Of note, NHIF restricts reimbursement of DMDs to clinically definite MS, therefore those medications may not be prescribed in clinically or radiologically isolated syndrome with financial support.

### Setting of database

The details of NEUROHUN database have been published elsewhere [19]. Briefly, we used data submitted to the NHIF by healthcare providers with contract, including all public hospitals, contracted private hospitals, and outpatient specialist services, between 1st January 2004 and 31st December 2016. To ensure personal data protection, the original social security identifiers were centrally anonymized for our database by the NHIF. This encrypted identifier was used for record linkage in our analysis. For each individual, basic patient features (year of birth, gender, postal code of residence) and data on all hospitalizations, as well as of all outpatient specialist care or diagnostic services used during this 13-year period were available. Those data included date, specialty and institution of provider, submitted interventions and diagnosis. Our database covers all specialist inpatient and outpatient services but does not include reports from primary care by general practitioners, which are submitted separately to the NHIF and therefore here could not be evaluated. Date of death–if occurred during the observational period–was provided by the Central Statistical Bureau of Hungary and linked to the subjects.

For each submitted claim towards the NHIF the care provider has to declare at least one diagnosis using the 10th International Classification of Diseases (ICD-10) codes, and our database contained all primary and secondary diagnoses for each claim. For hospitalized inpatients, the number of ICD-10 diagnoses assigned for each patient and report are not limited, but the category of diagnosis (i.e. basic disorder, primary diagnosis reported for reimbursement, accompanying disorder, complication, cause of death) should be coded. As for the outpatient services, only the main diagnosis is reported.

The NHIF provided anonymized data on all subjects, who has received at least once a diagnostic code of neurological (ICD-10 codes: G00-G99) or cerebrovascular (I60-I69) diseases, benign, uncertain or malignant neoplasms of the meninges and the central nervous system (D32-33, D42-43, C69-72) and some unspecified neurological symptoms (headache: R51; pain, unspecified: R52; malaise and fatigue: R53, syncope and collapse: R55; convulsions, not elsewhere classified: R56) between 1st January 2004 and 31st December 2016. This database contained healthcare consumption data of 4.29 million individuals. Of them, we have identified all subjects who were given–at least once–the diagnostic code of MS (G35) as primary or secondary diagnosis, resulting an “MS-database” of nearly 34,400 subjects.

For outpatient pharmacy refills, the commercial name, the chemical name, the ATC code and the amount of the drug, the ICD-10 code of the indication (limited to one single code), the specialty of the prescribing physician and the date of refill are registered and linked to the patient. Data of pharmacy refills were provided for our database only after 1st January 2010. Over-the-counter medication use is not registered in this database.

Data have been provided by NHIF after centrally encrypting the original personal identifier number, thus ensuring anonymity, so consent was not obtained from subjects. We used this encrypted identifier for record linkage between the clinical and pharmacy databases. The study was approved by the Ethics Committee of Semmelweis University, Budapest (Approval No: SE TUKEB 88/2015), and data were handled in accordance with personal data protection regulations.

### Algorithm for data extraction and case definition

In ICD-10 multiple sclerosis has a unique code (G35), which is widely used in clinical practice, and it is the only code authorized to be indicated on prescriptions of DMDs in Hungary.

Based on previous works of Marrie et al. on validity of case-definitions of MS using regional administrative data [20], we considered as MS-cases all individuals who fulfilled all of the following 3 criteria:

- had received ICD-10 diagnostic code of MS (G35) at least 3 times (at 3 separate medical contacts) during the observation period, whether inpatient or outpatient in any of the hospitals or any outpatient services of the country. To ameliorate specificity, we have ignored when diagnosis had been given on the occasion of laboratory, imaging, pathology or other diagnostic services used, as it is sometimes only a suspected diagnosis which justifies examination and not a confirmed one.
- at least one of these ≥3 claims for MS was submitted by a neurologist (hospitalization on neurology ward or use of neurological outpatient care), that we consider as a confirmation of the diagnosis.
- during the observational period, the patient received G35 code in at least 2 separate calendar years (not necessarily consecutive), to exclude patients who had been hospitalized for suspected MS which was finally ruled out.

### Validation of sensitivity of case definition versus clinical diagnosis and test of specificity

In order to examine the reliability of the above detailed MS case definition, we performed a two-way validation of administrative cases compared to clinical diagnosis in medical documentation held by our department. As the administrative database is anonymized, this validation process had to be restricted only to patients treated by our department.

First we have chosen two 2-month-long periods (between 1st May 2011 and 30th June 2011, and between 1st May 2014 and 30th June 2014). With the help of the local integrated hospital healthcare IT system (MedSol) we identified all subjects who were managed during that period at our neurological department and were given an ICD-10 code of MS, either in the inpatient or outpatient setting, with the exclusion of diagnostic (electrophysiology, ultrasound) and paramedical (physiotherapy, neuropsychology) services.

In the next step, we searched the NEUROHUN database for those individuals whose claim was submitted by our neurological services during the periods in question and received a diagnostic code of G35. We attempted to find a match of our patients whose neurological documentation is held by Semmelweis University, Budapest, with the subject in the anonymized administrative database by their admission and discharge date, sex, year of birth, postal code and service provider code. After successful match, we verified the clinical diagnosis of each patient by reviewing their hospital documents. We considered a patient having MS when the McDonald criteria of 2005 [21] were fulfilled until 31th December 2016. When clinical data suggested MS but the results of the ancillary examinations were not available for definitive diagnosis, we considered the subject not having MS.

In the final phase, we applied our MS case definition on all subjects retrieved from NEUROHUN whose claim had been submitted during the time periods in question and checked the concordance of administrative and clinical diagnosis.

When testing the sensitivity of the case definition, we have used the results of this cohort where true positive and false negative cases were identified based on the medical documentation as gold standard. On the other hand, specificity could not be calculated correctly using the results above, as we were examinig those consecutive patients who have had received at least once the diagnosis of G35 in a given period and thus are at risk of having MS, hence the low number of true negative cases. Instead, we have applied the method used by Bezzini et al. [22] with some necessary modifications. First, from the original patient-database including 4.29 million subjects, we created an other cohort of individuals who were presumably not affected by MS. This true negative reference cohort was defined by: never undergone cranial or spinal cord MRI and never received prescription of any drug with the code of G35 on the prescription during the observational period. Then, we linked this true negative cohort with those subjects who fulfilled our case definition of MS and analyzed the number and proportion of overlapping individuals, regarded as being false positive.

### Identification of subjects from the pharmacy database

From the pharmacy dispension database, first all subjects were identified each year between 2010 and 2016 who have at least once received any pharmacy refill with a diagnosis code of G35 on the prescription. Then, a second search in the database identified those individuals who had at least one pharmacy refill for any DMD available in that period in Hungary, specifically: intramuscular interferon-beta-1a (Avonex), subcutaneous interferon-beta-1a (Rebif), interferon-beta-1b (Betaferon, Extavia) and glatiramer-acetate (Copaxone), oral dimethyl-fumarate (Tecfidera), fingolimod (Gilenya) and teriflunomide (Aubagio), intravenous natalizumab (Tysabri) and alemtuzumab (Lemtrada). Pharmacy refill records then were linked to reports of medical service providers by the encrypted identifier.

### Calculation of prevalence and incidence

The date of the first medical encounter (inpatient or outpatient service) with ICD-10 code G35 assigned as primary or secondary diagnosis was considered the date of establishing diagnosis of MS. These patients were counted as incident cases for that year.

Thus, patients already diagnosed with MS before 2004 will also appear as incident cases at the time of their first medical encounter for MS during the observational period. To minimalize this bias, we considered incidence and prevalence data only from 2010 (allowing a 6-year “run-in” period), the starting year of pharmacy refill data availability. Similarly, as our 3^rd^ criteria for establishing MS from the administrative database was the 2 calendar year rule, patients who first appeared in the database with G35 code in 2016, although could turn to be true MS patients in subsequent years, administratively would appear as not MS patients, since the third criteria of our definition cannot be met in one year. Therefore we decided not to consider incidence and prevalence data of 2016. However, number of incident and prevalent patients, crude incidence and prevalence of 2004–2009 and 2016 are available and we present these as supporting information.

Prevalence was estimated as incident cases added to prevalent cases from the previous year, subtracting patients died during the year. Crude incidence and prevalence were calculated per 100,000 inhabitants, for women, men and both sexes, using corresponding data of the latest nationwide census in 2011 [17]. We have estimated age-adjusted standardized incidence and prevalence as well, using direct standardization method, based on the European Standard Population of 2013 as reference [23, 24].

Confidence intervals for the prevalence and incidence rates were calculated using the gamma distribution [25, 26]. The significance of the trends was tested with linear regression, *p*-value was considered significant if ≤0.05. The goodness of fit of the linear regression models was tested using the Shapiro-Wilks test of normality. The calculations were conducted with the R programming language (version 3.6.2) using packages “epitools” and “asht”.

## Results

### Validation of case definition in our center

After searching the hospital IT-system (MedSol) we have identified 42 cases of inpatients (25 cases in 2011 May-June and 17 in 2014 May-June, altogether 40 individuals) and 517 cases of outpatients (231 cases of 166 subjects in 2011 May-June and 286 cases of 204 subjects in 2014 May-June, representing 291 individuals) who had received at least once the billing code of G35 in primary or secondary position. Of note, one inpatient from MedSol and one subject from the NEUROHUN could not been perfectly matched with any of the other database (we suspect here a mistype of year of birth, as other parameters and all dates of medical encounters are matching). This subject of 2011 in NEUROHUN is not fulfilling our case-definition of MS and the medical documentation of the subject of MedSol reveals seronegative neuromyelitis optica spectrum disease (NMOSD) as diagnosis. This patient was excluded from validation analysis.

Because of the overlapping between in- and outpatients, altogether it meant 309 individuals receiving the diagnosis of G35, summarized in Table 1. Of them, 275 MS-patients (89%) are–correctly–fulfilling our case definition, and 15 patients having other medical conditions–correctly–do not fulfil our case definition (however, they have at least once received code G35 and one of them had a second neurological attack in 2017 fulfilling McDonald criteria and becoming MS-patient that time). One MS-patient did not fulfil our third criterion as he received G35 more than 3 times only in one single calendar year, meaning he discontinued medical follow-up for his condition not only in our center but in the whole public health care. He can be considered as “false-negative”. We found 18 subjects (5.8%) who were administratively classed as MS (“false-positive” cases) but medical documentation reveal that they have other illness after initially considered as MS or examinations are not completed or missing so establishing diagnosis of MS is hindered. Thus, the sensitivity of our case definition turned out to be 99%, while the positive predictive value is 94%.

**Table 1:** Summary of patients managed in our department and received G35 in any diagnosis position in May-June 2011 and May-June 2014.

ALL: 309 patients	administrative case-definition of MS is fulfilled number of patients *(percentage)*	administrative case-definition of MS is not fulfilled number of patients *(percentage)*
medical documentation states MS	275 *(89%)*	1 *(0*.*3%)*
medical documentation is not decisive or states another diagnosis	18 *(5*.*8%)* (5 NMOSD, 1 neurodegeneration with brain iron accumulation, 2 small vessel disease, 1 leukodystrophy, 2 not organic symptoms, 1 PSP, 1 polyneuropathy, 1 Pompe-disease, 1 unknown white matter disease with epilepsy, 3 patients not finishing examinations)	15 *(4*.*9%)* (1 will be MS in 2017, 1 BPPV, 1 unilateral abducent nerve palsy, 1 Leber’s optic neuropathy, 1 partial epilepsy, 2 not organic symptoms, 1 cerebral vasculitis, 1 Bell's palsy, 1 myelitis transversa, 1 cervical disc herniation, 1 paraesthesia of lower limbs, and 3 patients not completing examinations)

NMOSD, neuromyelitis optica spectrum disease; PSP, progressive supranuclear palsy; MS, multiple sclerosis; BPPV, benign paroxysmal positional vertigo.

To calculate specificity of the case-definition, we have created a true negative reference cohort as discussed above. The number of individuals, who have never undergone cranial nor spinal MRI and have never had prescription of any drugs for MS turned out to be 3,223,001. This cohort was then linked to the cohort of MS-patients: of 14437 subjects 1023 (7%) were overlapping and thus regarded as false positive. It meant a specificity of >99%. Worth of note, that this setting is not applicable for calculation of sensitivity, as the number of false negative patients remains unknown.

### Crude prevalence and incidence between 2010–2015

From 2004 to 2016, altogether 14437 people met our administrative case definition of MS. As discussed above, we allowed a 6-year-long “run in” period and not considered data until 2010 and those of 2016. The number and gender distribution of incident and prevalent cases between 2010 and 2015 are shown in Table 2. During that period, the annual crude prevalence of MS has increased continuously from 109.3/100,000 to 130.7/100,000, mirroring a rise from 150.8/100,000 to 179.5/100,000 among women and from 63.3/100,000 to 76.8/100,000 among men. This growing trend was significant (*p*-value of linear regression model <0.05 for all the three datasets). The ratio between women and men living with MS remained invariably 2.6 during these years.

**Table 2:** Crude incidence and prevalence of MS in Hungary between 2010 and 2015.

	number of incident cases (women/men)	number of prevalent cases (women/ men)	woman/man ratio of prevalent cases	woman/man ratio of incident cases	total crude incidence *(95% CI)*	crude incidence among women *(95% CI)*	crude incidence among men *(95% CI)*	total crude prevalence *(95% CI)*	crude prevalence among women *(95% CI)*	crude prevalence among men *(95% CI)*
2010	703 (500/203)	10859 (7872/2987)	2.6	2.5	7.1 *(6*.*6–7*.*6)*	9.6 *(8*.*8–10*.*5)*	4.3 *(3*.*7–4*.*9)*	109.3 *(107*.*2–111*.*4)*	150.8 *(147*.*5–154*.*2)*	63.3 *(61*.*1–65*.*6)*
2011	616 (429/187)	11338 (8206/3132)	2.6	2.3	6.2 *(5*.*7–6*.*7)*	8.2 *(7*.*5–9)*	4.0 *(3*.*4–4*.*6)*	114.1 *(112*.*0–116*.*2)*	157.2 *(153*.*9–160*.*7)*	66.4 *(64*.*1–68*.*7)*
2012	653 (446/207)	11809 (8543/3266)	2.6	2.3	6.6 *(6*.*1–7*.*1)*	8.6 *(7*.*8–9*.*4)*	4.4 *(3*.*8–5*.*0)*	118.8 *(116*.*7–121*.*0)*	163.7 *(160*.*2–167*.*2)*	69.2 *(66*.*9–71*.*6)*
2013	625 (434/191)	12234 (8857/3377)	2.6	2.3	6.3 *(5*.*8–6*.*8)*	8.3 *(7*.*6–9*.*1)*	4.0 *(3*.*5–4*.*7)*	123.1 *(120*.*9–125*.*3)*	169.7 *(166*.*2–173*.*3)*	71.6 *(69*.*2–74*.*0)*
2014	592 (389/203)	12634 (9113/3521)	2.6	1.9	6.0 *(5*.*5–6*.*5)*	7.5 *(6*.*7–8*.*2)*	4.3 *(3*.*7–4*.*9)*	127.1 *(124*.*9–129*.*4)*	174.6 *(171–178*.*2)*	74.6 *(72*.*2–77*.*1)*
2015	538 (375/163)	12993 (9369/3624)	2.6	2.3	5.4 *(5*.*0–5*.*9)*	7.2 *(6*.*5–8*.*0)*	3.5 *(2*.*9–4*.*0)*	130.8 *(128*.*5–133*.*0)*	179.5 *(175*.*9–183*.*2)*	76.8 *(74*.*3–79*.*4)*
*p*-value^a^	N.A.	N.A.	N.A.	N.A.	0.018276*	0.011609*	0.258283	0.000003*	0.000006*	0.000002*

Crude incidence: new patients/100,000 inhabitants/year. Crude prevalence: number of living patients/100,000 inhabitants.

^a^*p*-value: *p*-value of trend significance test using linear regression. The *p*-value ≤0.05 was considered significant and is marked with asterisks*

N.A., not applicable; CI, confidence interval. Gamma confidence intervals were created using R version 3.6.2 with package “epitools”. The method is based on Daly [25].

On the other hand, the number of incident cases as well as crude total incidence has declined (the latter from 7.1/100,000 in 2010 to 5.4/100,000 in 2015, *p*-value = 0.018) with a smaller rise in 2012. The Shapiro-Wilks test indicated that linear models for the total crude incidence trend analysis may be inappropriate (*p*-value <0.05), thus results related to this model should be interpreted with caution. The crude incidence for women has also diminished from 9.6/100,000 to 7.2/100,000, showing a negative significant trend (*p*-value <0.05) together with the crude total incidence. The incidence among men has changed from 4.3/100,000 to 3.5/100,000, but the trend was not significant. The female/male ratio of incident cases is found to be between 1.9 and 2.5 during these years.

### Standardized prevalence and incidence between 2010–2015

Using the EU2013 standard European population as reference, age adjusted standardized prevalence of MS in Hungary has gradually increased from 105.2/100,000 (147.3 for women and 60.3/100,000 for men) in 2010 to 127.2/100,000 (175.6 for women and 74.7/100,000 for men) in 2015. These positive trends were significant (*p*-value <0.05). Yearly data are shown in Table 3.

**Table 3:** Age-adjusted standardized prevalence and incidence of MS in Hungary.

	Age-adjusted standardized prevalence, total *(95% CI)*	Age-adjusted standardized prevalence, women / men *(95% CI)*	Age-adjusted standardized incidence, total *(95% CI)*	Age-adjusted standardized incidence, women / men *(95% CI)*
2010	105.2 *(103*.*2–107*.*3)*	147.3 / 60.3 *(144*.*0–150*.*6) / (58*.*1–62*.*5)*	6.7 *(6*.*2–7*.*2)*	9.5 / 3.9 *(8*.*7–10*.*4) / (3*.*4–4*.*4)*
2011	109.9 *(107*.*9–112*.*0)*	153.3 / 63.4 *(150*.*0–156*.*7) / (61*.*1–65*.*7)*	5.9 *(5*.*4–6*.*4)*	8.1 / 3.7 *(7*.*4–8*.*9) / (3*.*2–4*.*3)*
2012	114.6 *(112*.*5–116*.*7)*	159.6 / 66.3 *(156*.*2–163*.*0) / (64*.*0–68*.*7)*	6.2 *(5*.*7–6*.*7)*	8.5 / 3.9 *(7*.*7–9*.*3) / (3*.*4–4*.*5)*
2013	119*(116*.*9–121*.*2)*	165.6 / 68.9 *(162*.*2–169*.*1) / (66*.*5–71*.*3)*	6 *(5*.*5–6*.*5)*	8.2 / 3.7 *(7*.*5–9) / (3*.*2–4*.*3)*
2014	123.4 *(121*.*2–125*.*6)*	170.7 / 72.2 *(167*.*2–174*.*3) / (69*.*8–74*.*3)*	5.7 *(5*.*2–6*.*2)*	7.4 / 3.9 *(6*.*7–8*.*2) / (3*.*4–4*.*5)*
2015	127.2 *(125*.*0–129*.*4)*	175.6 / 74.7 *(172*.*0–179*.*2) / (72*.*2–77*.*2)*	5.1 *(4*.*7–5*.*6)*	7.1 / 3.1 *(6*.*4–7*.*9) / (2*.*7–3*.*7)*
*p*-value^a^	0.000001*	0.000002* / 0.000001*	0.015974*	0.012586* / 0.222197

Age standardization was performed using the 2013 European standard population. Incidence and prevalence are expressed as rate/100,000 population.

^a^*p*-value: *p*-value of trend significance test using linear regression. The *p*-value ≤0.05 was considered significant and is marked with asterisks*

CI, confidence interval. Gamma confidence intervals were created using R version 3.6.2 with package “asht”. The method is based on Fay & Feuer [26].

As for age adjusted standardized annual incidence of MS, it remained quite stable among men between 2010 and 2014 (3.7–3.9/100,000) and slightly diminished only in 2015 (3.1/100,000), without showing a significant trend. Interestingly, for age-adjusted standardized incidence among women we could observe a significant negative trend (*p*-value = 0.0125) with a slow decline from 9.5/100,000 in 2010 to 7.1/100,000 in 2015. The standardized incidence for both sexes was 6.7/100,000 in 2010, then decreased in 2011, followed by a rise in 2012 and a gradual diminution afterwards to reach 5.1/100,000, altogether representing a significant negative trend (*p-*value = 0.016), however, the Shapiro-Wilks test indicated that linear models for the total age-adjusted standardized incidence trend analysis may be inappropriate (p<0.05), thus results related to this model should be interpreted with caution.

### Drug dispension data

Between 2010 and 2015 the number of patients who refilled any (except for over-the-counter) drug in that year with an indication for MS–i.e. with a diagnosis code of G35 on the prescription–has increased from 6162 to 7399, see Table 4. Of them, the proportion of those who do not fulfil our case definition of MS is dropping from 15% to 12.5% as the number of these subjects remains quite stable around 800. We have to note that family doctors and any specialist can prescribe drugs with the code of G35, but some of these medicines are only reimbursed when prescribed by a neurologist or family doctor on the recommendation of a neurologist.

**Table 4:** Yearly drug dispension data.

	Nr of patients refilling ANY drug with G35 diagnosis in that year (*those who fulfil our case definition / those who do not*)*	Nr of patients refilling any of the 11 DMD and fulfilling our case definition of MS / Nr of patients refilling any of the 11 DMD and NOT fulfilling our case definition of MS*	Nr of patients refilling any DMD first time and fulfilling our case definition of MS / Nr of patients refilling any DMD first time and NOT fulfilling our case definition of MS	ratio of MS patients who received DMD that year
2010	6162 (*5358/804*)	2089 / 23	2089^a^ / 23^a^	0.19
2011	6326 (*5535/791*)	2746 / 34	828 / 33	0.24
2012	6539 (*5758/781*)	2969 / 42	392 / 38	0.25
2013	6671 (*5920/751*)	3108 / 52	357 / 48	0.25
2014	7130 (*6314/816*)	3482 / 60	500 / 56	0.28
2015	7399 (*6573/826*)	3808 / 70	466 / 63	0.29

*One patient can appear in more than one year.

^a^Drug dispension data are available only from 2010 therefore all patients will appear as “first time refill” this year.

In order to focus on a more specific patient group, we have searched those subjects who have at least once refilled any of the 11 DMDs listed earlier, and therefore considered as having relapsing-remitting MS by the prescribing clinician. The number of these patients has considerably grown between 2010 and 2015 (from 2089 to 3808). When applying our case-definition of MS on them, the proportion of those who do not fulfil it remains under 2% each year. The number of previously treatment-naive, newly DMD-treated patients (Table 4 fourth column) cannot be taken into account in 2010 (as the pharmaceutical database is available between 2010 and 2016 so every patient treated in 2010 will appear as new that year), but after a high number in 2011 there is a drop in 2012 and 2013, followed by a rise in 2014. This is in line with the launch date of some DMDs on Hungarian market (natalizumab in 2010; fingolimod, dimethyl-fumarate and teriflunomide in 2014).

When comparing the number of prevalent MS-cases to that of subjects refilling DMD, we can assess the proportion of DMD-treated MS-patients. This ratio significantly rose from 0.19 in 2010 to 0.29 in 2015 (Table 4), the *p*-value of trend analysis with linear regression is 0.0051.

## Discussion

In this study we present our work on determining an administrative case definition of MS, followed by a two-step case-certification based on drug dispense records and a local cohort of patients. Given the lack of national MS patient registry, our main aim was to estimate the number of individuals living with MS in Hungary, as previously only regional data were published on epidemiology of MS [11–16]. In their most recent work, based on the patient register of one university MS-center, Biernacki et al. [16] reported the standardized prevalence being 101.8/100,000 population (53.9 and 144.8/100.000 for men and women, respectively) in 2019. Meanwhile, in MS Barometer 2015 [4] the Hungarian MS Society has reported that the estimated number of MS-patients countrywide was 8000, and in the review of Global Burden of MS [5] the number of subjects living with MS in Hungary is estimated to be between 5927 and 7480 in 2016, which both implicate a lower prevalence. Our study reveals a significantly higher adjusted prevalence (119/100,000 in 2013 and 127.2/100,000 in 2015) than reported beforehand, showing a continuous rise between 2010 and 2015. The difference between the regional and administrative nationwide data could be the result of using different methods, for example that chronically disabled or bedridden patients may not visit regional university MS-center and thus are not registered there (recruitment bias), which is plausible regarding the rather low average EDSS score (2.8 points ±2.44) and high percentage of DMD-treated subjects (74.28%) [16]. Other regional factors (number and availability of neurologists, ethnic and age distribution of population) can also play a role.

Hungary is part of the Central European region which used to be considered to show medium risk for MS. However, recent works from the area reveal a somewhat higher prevalence (i.e. above 100/100,000 population). Those include a study from Austria using administrative data [27], which found an average crude prevalence at 158.9/100,000 and average crude incidence rate at 19.5/100,000 person-years in 2011–2013. In Poland, a research based on a regional MS-registry showed that standardized prevalence was 106.6/100,000 in 2014 in Central Poland [28]. Later, prevalence was investigated in an other Polish region as well, using data from the same patient registry system, and there the standardized prevalence turned out to be 108.5/100,000 [29]. In Croatia, after merging 3 databases of public health care and that of the MS-patient society, the crude prevalence was found to be 143.8/100,000 in 2015, much higher than previously estimated [30]. Daltrozzo et al. [31] analyzed the database of ambulatory claims held by Bavarian Association of Statutory Health Insurance Physicians. They demonstrated that in Bavaria the standardized prevalence of MS increased from 166.7/100,000 to 266.36/100,000 between 2006 and 2015, the latter being among the highest rates worldwide. Our results are in line with those higher prevalence rates in Central Europe, even if direct comparison is hampered by the use of different methods and lack of standardization. The highest prevalence rates (above 200/100,000) worldwide are reported in Canada, the United Kingdom and Northern Europe [32–34].

Rising prevalence of MS is observed worldwide [3, 35, 36] and is at least partly related to longer disease duration, which could be caused by earlier age at onset of disease, earlier diagnosis and especially higher age at death with improved survival [32, 37, 38].

In our study, the ratio of female and male prevalent cases remains 2.6 during the observational period, and the women/men ratio of incident cases was between 1.9 and 2.5. These results are also in line with the results of other groups [39–41].

As long as we know, there were no studies focused on the incidence rate of MS in Hungary in the past 20 years. Here we found that the standardized total incidence rate was between 6.7 and 5.1 (/100,000) from 2010 to 2015, showing a significant negative trend, in parallel with the changes of standardized incidence rate in women (between 9.5 and 7.1/100,000), while that of men remained stable (3.1–3.9 /100,000) during these years. Similar trends in annual incidence–showing lack of increase or even decrease in total and some decrease among women–were found for example in different counties of Canada by Marrie et al. [20], Rotstein et al. [32], and Kingwell et al. [40], furthermore in the United Kingdom by Mackenzie et al. [33] but several studies reveal rise in incidence rates during the last decades [34, 42, 43] and especially among women [9, 44], the latter reaching rates higher than 10/100.000 in Northern Europe. These differences in trends of incidence rates are difficult to explain, but environmental, socio-economic and genetic factors might play a role. Also, the changes in coding policies, like giving diagnosis of clinically or radiologically isolated syndrome instead of MS until the actual diagnostic criteria are not fulfilled may contribute to the decline in incident administrative cases.

In the past decade, the use of administrative data for examining epidemiology of diseases and comorbidities has been more and more frequent. Regarding MS, among the first works aiming to determine an administrative MS case definition was carried out by Culpepper et al. investigating the Veterans Health Administration databases in the USA [45] and Marrie et al. in Canada [20] using health insurance claims data in the province of Manitoba. In the latter, the following MS case definition was established and validated: ≥3 hospital, physician, or prescription claims for MS. Since then it was widely applied to determine prevalence in other regions [40, 46], and to investigate the epidemiology of comorbidities in MS [47, 48]. Later, other Canadian researchers used a different administrative case definition which was one hospitalization or at least 5 physician billings for MS over 2 years [49]. These two administrative definitions were validated on medical records and compared in a Canadian county [50]. It was found that the Marrie-definition has a sensitivity of 99.5%, a specificity of 98.5%, a positive predictive value of 99.5% and a negative predictive value of 97.5%, altogether presenting a better performance. Recently, Culpepper and Marrie both have participated in the United States Multiple Sclerosis Prevalence Workgroup who published [51] on testing the performance of different administrative algorithms for identifying MS cases. They recommended the definition of ≥3 MS-related claims in any combination of inpatient, outpatient, or DMT use within 1 calendar year to become standard.

In their consecutive works on prevalence and incidence of MS in Tuscany, Bezzini et al. [22, 52, 53] used and validated the administrative case definition of meeting at least one of the following 4 criteria: at least one hospital discharge record with MS diagnosis or one active payment exemption for MS or at least 2 prescriptions for MS-specific drugs or MS diagnosis in home and residential long-term care data. The sensitivity of this definition turned out to be 98%, while specificity was 99%.

A recent study of Roux et al. [54] analyzed nationwide health administrative data of France between 2010–2015 and used the following case definition: at least one reimbursement for a DMD or an active status of “long-term disease” for MS or at least one hospitalization with a discharge diagnosis of MS. Salhofer-Polanyi et al. [27] examinig Austrian healthcare data defined as MS-patient who had at least 1 prescription for DMD or at least one hospitalisation with discharge diagnosis of MS during the 4-year-long observational period. Other examples of administrative MS-definitions include that of Höer et al., who considered a subject having MS if he or she had ≥1 claims for MS documented by neurologist or psychiatrist, or ≥1 prescription of DMD [55]. Daltrozzo et al. also investigated Bavarian healthcare database and used an MS case-definition similar to ours but less strict: G35 given at least in two separate quarterly periods, coded at least once by neurologist [31].We had to make some modifications on the case definition of Marrie as we did not have access to the records of family doctors, and the medication refill database was available only from 2010, not for the entire period of the claim database. We aimed for a more conservative estimation of MS-cases and added a temporal restriction (G35 occurring in minimum 2 calendar years) to exclude when MS is only the suspected diagnosis of hospitalization or consultation and diagnostic workup reveals another medical condition. Since even non-DMD-treated MS patients are advised to see a neurologist once a year, they are probably captured by this definition, as the above mentioned French study [54] found that over 6 years of observation 75.1% of MS-patients had visited a neurologist at least once a year. In order to minimize false positive MS-diagnosis, we also added the criterion that at least one code of G35 should be given by a neurologist that we considered as “confirmation” of the diagnosis.

The case certification and therefore the validation of our case definition was performed on the entire patient cohort receiving diagnosis of G35 of two randomly selected 2-month-long time-intervals in a single University center. We identified 309 individuals as possible MS-patients based on the billing code of G35 in primary or secondary position (Table 1). When applying the case-definition and comparing that with the diagnosis found in medical records (considered as gold standard), it turned out that the administrative definition performed correctly in 93.9% of the entire cohort of “MS-suspect patients”. The administrative MS-diagnosis turned out to be “false-positive” in 18 cases (5.8%) in those who fulfilled administrative MS case definition but clinically MS diagnosis was not possible to determine (3 subjects whose evaluation was not finished or the results were not available) or the subject had other medical condition. Worth of note that of them, 5 patients suffered from NMOSD which both clinically and radiologically can mimic MS and used to be considered as an MS-subtype before the discovering of anti-aquaporin-4 antibody. Further 2 subjects had been for years considered as having MS before this diagnosis was revised (neurodegeneration with brain iron accumulation and small vessel disease). We have identified only 1 “false-negative” MS-case who did not fulfil the criterion of having claim for MS in 2 separate calendar years because of refusing medical follow-up. Thus, the sensitivity of the administrative case-definition was 99%. The specificity was tested with the help of an administrative “true negative” cohort and turned out to be >99%.

When analyzing drug dispension data between 2010 and 2015, we first focused on DMD refills: these are highly specific for treating MS and their prescription is centralized and regulated in Hungary, so we had speculated that among these subjects the probability of having MS is high and they would also serve as a validation cohort. Indeed, each year only 1–2% of DMD-refilling patients do not fulfil our administrative case definition. A continuous rise of number of DMD-treated patients can be observed with nearly doubling in 5 years, that is partly explained by the introduction of new DMDs in the Hungarian market and the growing number of MS patients. The ratio of DMD-treated patients was relatively low in 2010 (0.19) and significantly rose to reach 0.29 in 2015. It still can be considered low, but according to pharmaceutical industry estimations [56] considerable differences exist even among European countries, with a proportion of DMD-treated patients as high as 69% in Germany and as low as 13% in Poland in 2013. However, our data are in line with some other publications: Kingwell et al. [40] reported–based on administrative data–that 29% of MS patients had received at least once a prescription for a DMD between 1991 and 2010 in British Columbia. Höer et al. also worked with administrative data and definition [55] and found that 50.5% of MS patients had at least 1 prescription of DMD in 2009. According to data of Central Italy [22], 41% of MS-patients received at least 2 prescriptions for DMD in 2011. Differences in the proportion of DMD-treated MS patients are probably multifactorial: besides national regulations on prescription (for example in Hungary DMD is not reimbursed in clinically isolated syndrome), genetic and environmental issues also play role, for example in affecting the proportion of benign or primary progressive MS.

The strengths of our study include the use of nationwide administrative healthcare data with a 13-year-long observational period, allowing the assessment of temporal trends. The setting of the database enabled that we could consider the presence of G35 not only as primary diagnosis but in any position if recorded at medical encounter. We validated our administrative MS case definition on the medical records of 309 subjects who had received G35 during a given time period in our neurological department, and by comparing administrative and medical diagnosis we found that 93.8% of the patients were classed correctly. Both sensitivity and specificity of the case definition were 99%. Furthermore, with the help of an independent database of pharmacy refills we could re-assess the performance of this case definition, as of the individuals who refilled at least once at least one DMD 98% fulfilled the MS case definition. It made possible the estimation of the proportion of DMD-treated MS-patients as well.

We have to consider some limitations of our study: the work with administrative health data implicate the lack of clinical details of patients, for example, there was no information on which diagnostic criteria had been used when establishing and coding diagnosis of MS, neither on clinical subtype (relapsing-remitting, progressive) or regional differences, which could help to understand the relatively low proportion of DMD-treated individuals. Also, we have to keep in mind the possible errors of coding that was found as high as 4% in the cohort of Salhofer-Polanyi in Austria [27], and issues of diagnostic accuracy, the latter being not exceptional in MS given the nature of the disease [57].

In our setting, we did not have access to data of health service supplied by family doctors, that would help to better estimate the proportion of benign and severely handicapped MS-patients who may have not seen a neurologist during the 13 years of investigation and therefore may be underestimated. It could have explained some of the refilling of non-DMD drugs with code G35 by subjects not fulfilling our criteria. Indeed, the French study [54] has revealed that 13% of subjects with MS had not been seen by neurologist (neither public, nor private specialist) during the 6-year-long observational period, and these patients were older and had a longer disease duration. These patients also could at least partly contribute to explain the reduced incidence rates in our study.

Furthermore, the third criterion of our administrative case definition might seem to be too strict, but our primary aim was to minimize the risk of including people without MS and assumed that the majority of MS-patients would have some kind of medical encounter for their condition in at least 2 years of 13. A similar criterion was applied when the prevalence of Parkinsons’s disease was estimated with use of the same database [58] and that administrative case-definition also performed well. However, it can underestimate the incident cases of the last years of the observation period, as the two apparition of G35 required in two different calendar years are not necessarily consecutives and therefore may need several years to fulfil this criterion.

In summary, we have established and validated an administrative case-definition of MS, and first used it on the database of Hungarian National Health Insurance Fund that contains all in- and outpatient medical encounters in public healthcare between 2004–2016, and secondly, on the independent database of outpatient pharmacy refills between 2010–2016. Our findings highlight that MS prevalence is significantly higher in Hungary (127.2/100,000 in 2015) than previously reported and is growing, while incidence is relatively low and shows a decline. Further follow-up and investigation is needed to understand this unusual trend of the latter. The proportion of DMD-treated MS-patients was rather low (0.29 in 2015) that may partly be due to special restrictions of their prescription.

## Supporting information

S1 File (XLSX)

S2 File (XLSX)
